# Light-enhanced electrical behavior of a Au/Al-doped ZnO/p-Si/Al heterostructure: insights from impedance and current–voltage analysis

**DOI:** 10.1039/d3ra06340b

**Published:** 2023-09-29

**Authors:** Majdi Benamara, Kais Iben Nassar, Sonia Soltani, Afef Kallekh, Ramzi Dhahri, Hassen Dahman, Lassaad El Mir

**Affiliations:** a Laboratory of Physics of Materials and Nanomaterials Applied to the Environment, Faculty of Sciences of Gabes, University of Gabes Erriadh 6079 Gabes Tunisia majdibenamara1@gmail.com; b Department of Physics, I3N-Aveiro, University of Aveiro 3810-193 Aveiro Portugal; c Department of Physics, College of Science and Arts, Qassim University Dariyah 58251 Saudi Arabia; d Department of Mathematics, College of Science and Art Muhyl Assir, King Khalid University Abha 61413 Saudi Arabia

## Abstract

In this study, we meticulously deposited an Al-doped ZnO nanoparticle thin film on a p-type silicon substrate using the precise sputtering method. We conducted a comprehensive exploration of the film's structure, morphology, and optical properties. X-ray diffraction (XRD) confirmed its polycrystalline wurtzite configuration with a dominant (002) orientation. High-resolution scanning electron microscopy (SEM) and atomic force microscopy (AFM) revealed a uniformly textured surface adorned with densely packed nanoparticles. Regarding optical properties, the Al-doped ZnO thin film exhibited exceptional transmittance exceeding 80% across visible and near-infrared spectra. Moving on to electrical characteristics, we assessed the Au/Al-doped ZnO/p-Si/Al heterostructure under dark and illuminated conditions. Through current–voltage (*I*–*V*) and impedance measurements, we observed significant improvements in conductivity and performance under illumination. Notably, there was an increase in current conduction and a reduction in impedance, highlighting the advantages of illumination. Collectively, these findings emphasize the promising potential of the Au/Al-doped ZnO/p-Si/Al heterostructure, particularly in the realms of optoelectronic devices and photovoltaics. With its ability to efficiently mobilize charges and adeptly assimilate light, this heterostructure stands as a frontrunner for transformative applications in these technologically vital domains.

## Abstract 2

In this study, we meticulously deposited an Al-doped ZnO nanoparticle thin film on a p-type silicon substrate using the precise sputtering method.

## Graphical abstract

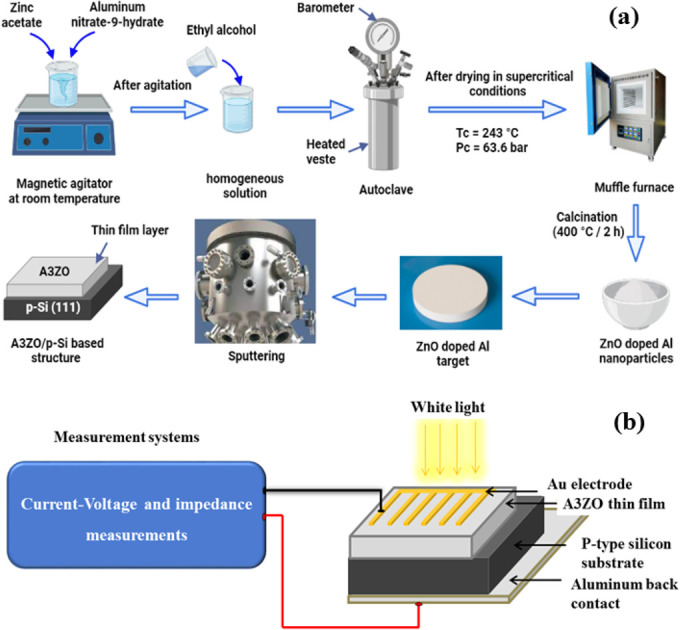

## 1. Introduction

High optical transparency and high electrical conductivity are two essential characteristics of transparent conducting oxides (TCOs), a class of materials widely employed in various optoelectronic applications such as phototransistors, liquid crystal displays, touch screens, optical detectors, and solar cells.^1–3^ TCOs are semiconductors that can be categorized as either p-type or n-type. Among these, zinc oxide (ZnO) stands out as an n-type TCO material, boasting a wurtzite hexagonal structure, a wide band gap of 3.38 eV, and a significant exciton binding energy of 60 meV.^4^ Due to its semiconductive and optical properties, ZnO films with various dopants have found extensive use in optoelectronic applications.^5,6^ Doped zinc oxide thin films have been prepared using several techniques, including plasma-assisted molecular beam epitaxy (PMBE), spin coating, chemical vapor deposition, radio frequency sputtering, and pulsed laser deposition (PLD).^7–11^ Among these methods, sputtering stands out as a promising approach, allowing the synthesis of doped ZnO nanoparticles in multiple layers, leading to polycrystalline films with a hexagonal structure under specific experimental conditions.^12^ Another avenue for enhancing the performance of ZnO-based optoelectronic devices involves surface modification. This technique entails the incorporation of metallic nanoparticles, such as aluminum (Al) or gallium (Ga) NPs, onto the device surfaces. These nanoparticles, thanks to their surface plasmon resonance (SPR) effect, have gained considerable attention in recent years for their potential to enhance light scattering and absorption in various devices, including solar cells, light-emitting diodes, and photodiodes.^13^

The SPR effect occurs when incident light with the appropriate frequency stimulates the free electrons within the nanoparticles, leading to a collective oscillation of these electrons under light illumination. This phenomenon results in an expansion of the optical path length of light within the semiconductor, ultimately boosting light absorption and device performance. SPR facilitates the interaction between light and free electrons at the interface, leading to electromagnetic field amplification and energy transfer from nanoparticles to the semiconductor layer.^14^ According to Mie theory, light scattering becomes prominent when the radius (*r*) of nanoparticles exceeds the wavelength of the incident light (2π*r* ≫ *λ*) for spherical particles.^15^ Notably, non-plasmonic nanoparticles lack the ability to significantly absorb visible light *via* SPR, distinguishing them from plasmonic counterparts. Therefore, the choice of nanoparticles is crucial in optoelectronic device design. Aluminum nanoparticles (Al NPs) are particularly favored due to their minimal absorption loss, especially in the UV region, and their strong scattering capabilities. Additionally, Al's lower inter-band transition threshold makes it highly advantageous for front-side decoration in devices. Recent findings emphasize the essential role of Al NPs in enhancing the photocurrent in photodetectors, primarily through the induction of an SPR effect and the generation of a strong electrical field in the near-field region, achieved by dispersing incoming waves widely.^16^

In this study, a thin film composed of Al-doped ZnO nanoparticles was deposited onto a p-type silicon (p-Si) substrate using the sputtering method. The resulting thin film was characterized by analyzing its structural, morphological, and optical properties. The structural analysis was carried out using X-ray diffraction (XRD) measurements. The XRD pattern of the Al-doped ZnO thin film revealed a polycrystalline wurtzite structure with a dominant (002) orientation. Morphological analysis was performed using scanning electron microscopy (SEM) and atomic force microscopy (AFM). The SEM image of the Al-doped ZnO thin film showed a uniform surface morphology with densely packed nanoparticles. Optical properties were evaluated using UV-visible-NIR transmittance measurements. The Al-doped ZnO thin film exhibited high transmittance (>80%) in the visible and near-infrared regions. Furthermore, the electrical properties of the fabricated Au/Al-doped ZnO/p-Si/Al heterostructure were evaluated under dark and light conditions at room temperature using current–voltage (*I*–*V*) and impedance measurements.

## 2. Experimental details

### 2.1. Sample preparation

In the first step, zinc oxide doped with 3% Al (A3ZO) nanopowder was synthesized using a sol–gel technique with 16 g of zinc acetate dihydrate [Zn(CH_3_COO)_2_·2H_2_O; 99%] as a precursor in 112 ml of methanol for the deposition of ZnO doped with 3% Al thin film on silicon substrate. An appropriate amount (6.93 g) of aluminum nitrate-9-hydrate [Al(NO_3_)_3_·9H_2_O] equivalent to a [Al/Zn] ratio of 0.03 was added after magnetic stirring at room temperature for 10 min. The solution was put into an autoclave and dried in supercritical ethyl alcohol (EtOH) (*T*_c_ = 243 °C, *P*_c_ = 63.6 bar). Additionally, the resulting nanopowder was annealed for two hours at 400 °C in air.

**Fig. 1:**
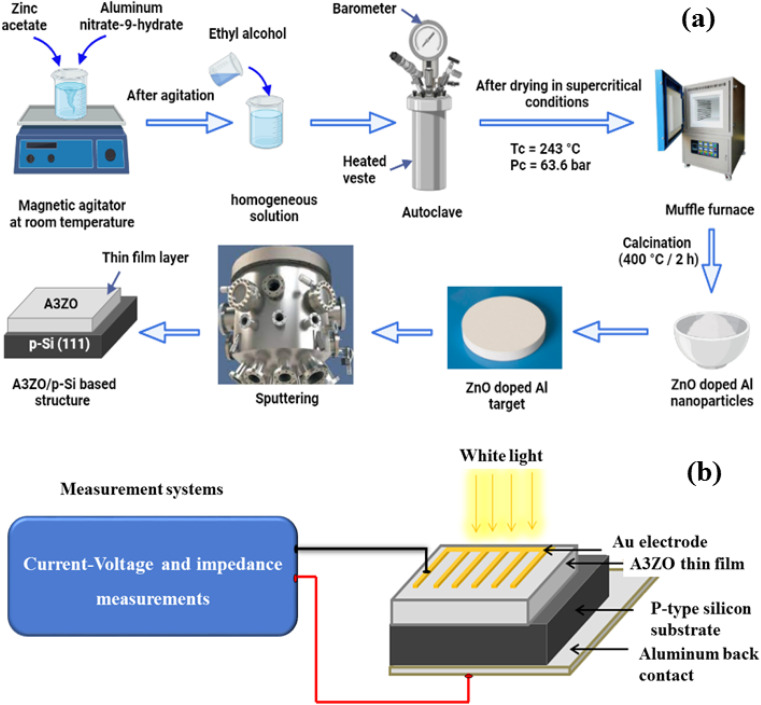
(a) Basic steps of the sample preparation and (b) measurement system.

The previously acquired A3ZO nanopowder was utilized in the second stage to sputter a thin coating onto a silicon substrate. Before allowing the argon gas with a high purity of 99.9999% to sputter, the sputtering chamber was evacuated to a base pressure of 6 10^−5^ Pa. The target-substrate distance was about 75 mm, and the rf power was kept at 25 W. The steps to the preparation of the sample were presented in the Fig. 1a. The top and bottom electrodes of the prepared sample, composed of gold (Au) and aluminum (Al), respectively, were deposited using the chemical vapor deposition (CVD) technique.

### 2.2. Characterizations

The crystal structure and particle size were investigated by X-ray diffraction (XRD, Bruker AXS D8 Advance). The morphology of our samples was investigated by scanning electron microscopy (SEM) equipped with EDX. SEM measurement was investigated by field-emission scanning electron microscopy (FE-SEM, S4800II, Hitachi, Japan). The surface morphology of A3ZO thin film was observed by atomic force microscopy (AFM, Benyuan Nanometer Instrument Co., Ltd). UV-visible-Near infrared spectrophotometer (Shimadzu UV-3101PC) in the wavelength range of 200 to 800 nm was used to perform the transmittance properties of our prepared structure. A Keithley 2400 source meter and an Agilent 4294A impedance analyzer, each with a signal of 50 mV, were used to measure the forward and reverse bias *I*–*V* and impedance, respectively. Both measurements were made at room temperature in both dark and light environments. The measurement system was implemented using a converter card and a microcomputer. The power supply (Model: 68938 Newport-Oriel Instruments) was powered by the solar simulator (Model: 6000 Q Series Lamp Housing Newport-Oriel Instruments), which served as the light source (Fig. 1b).

## 3. Results and discussions

### 3.1. Structural analysis

**Fig. 2:**
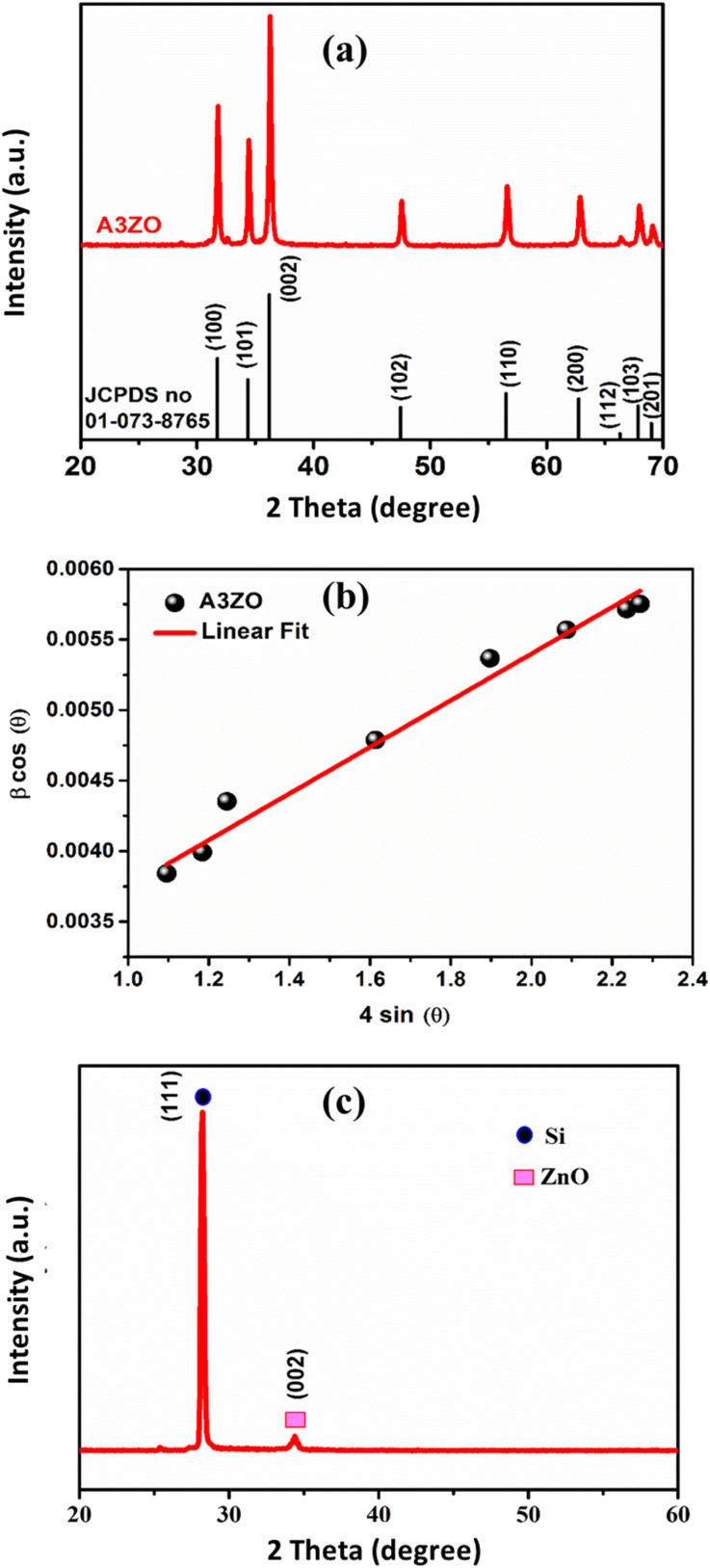
(a) X-ray diffraction pattern of 3 at% Al-doped ZnO aerogel nanoparticles and (b) Williams–Hall plot of A3ZO nanopowders. (c) XRD diffractogram of A3ZO thin film deposited on silicon substrate by sputtering.

The X-ray diffraction (XRD) patterns of the A3ZO nanopowders are presented in Fig. 2a. The presence of indexed peaks confirms the crystalline nature of both pure and doped ZnO, unequivocally demonstrating the characteristic hexagonal wurtzite structure with space group *P*6_3_*mc*. To delve deeper into the microstructural aspects of the material, we applied the Williamson–Hall model, which is governed by the following equation:1
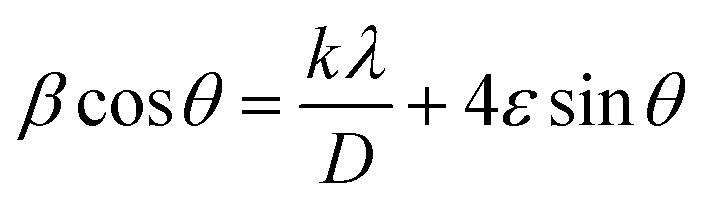
where *β* represents the full width at half maximum (FWHM), *θ* signifies the scattering angle, *k* stands as a material-specific constant derived empirically (0.9), *λ* denotes the wavelength of the X-rays (0.154 nm), *D* characterizes the average crystallite size, and *ε* denotes the micro-deformation or strain present within the crystal lattice. Through the construction of Williamson–Hall (W–H) plots, as depicted in Fig. 2b, with *β* cos *θ* against 4 sin *θ*, we could ascertain: the strain (*ε*): this parameter is deduced from the slope of the linear fit of the W–H plot, providing insights into the magnitude of micro-deformation or strain embedded within the material; the average crystallite size (*D*_WH_): this parameter is estimated from the intercept of the linear fit, offering a quantitative measure of the average crystallite size within the material. The lattice parameters (*a* and *c*) can be calculated from the highest maximum intensity and consequently the volume of the unit cell can be concluded through the following relationship:^17,18^

2 *V* = 0.866 × *a*^2^ × *c*

The density of the A3ZO samples can be calculated considering that the basic unit cell of wurtzite hexagonal structure contained strong ions using the following equation^19^3
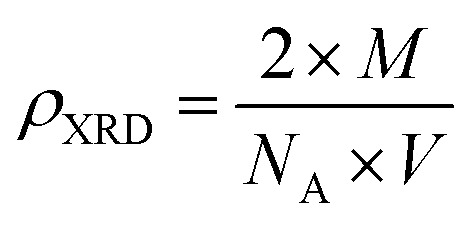
where *M*, *N*_A_ and *V* represent molecular weight, the Avogadro's number, and volume, respectively. Number 2 is the number of molecules in a unit cell of wurtzite hexagonal structure. From the calculated X-ray density, assuming all nanoparticles are spherical in shape, the specific surface area (S_XRD_) can be estimated from the following relationship:^20^4
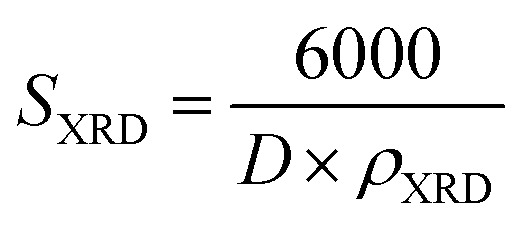
where 6000 is the spherical particles factor and *D* is the particle diameter calculated from W–H plots. Table 1 presents structural parameters of A3ZO samples.

**Table 1:** Structural parameters of the A3ZO nanoparticles

Parameters	*a* (Å)	*c* (Å)	*V* (Å^3^)	*D*_W–H_ (nm)	*ε* (10^−4^)	*ρ*_XRD_ (g cm^−3^)	*S*_XRD_ (m^2^ g^−1^)
Value	3.245	5.200	47.431	66	16.5	5.698	15.954

Fig. 2c displays the typical XRD of A3ZO thin film that was sputtered onto a silicon substrate. The measured diffraction peaks are oriented in the (111) and (002) directions after deposition on silicon. The primary peaks of silicon and zinc oxide are in directions (111) and (002), respectively. The silicon substrate's (111) plane is where the peak that was located at 28.22°. The hexagonal structure, or *c*-plane to the ZnO crystallites, corresponds to the densest packed plane, which results in the least surface energy that accounts for this (002) favored orientation. It is widely known that sputtered A3ZO thin films, particularly those with the (002) orientation, have a high degree of texture along the *c* axis perpendicular to the substrate surface. The polar character of the ZnO (002) plane, the low surface energy in this direction, and the strong growth kinetic along the *c* axis are all factors in the creation of this texture.^21^Fig. 2c displays the diffraction patterns of A3ZO films formed on p-type silicon substrates. Without a second phase, only the (002) diffraction peak is visible, and it is situated at 2*θ* = 33.26. This indicates that the films are polycrystalline, have a hexagonal structure, and favor a perpendicular *c* axis to substrate orientation. The slight deflection of the (002) peak suggested that there may be some residual stress inside the film, which is typical of films made by sputtering. However, certain ZnO films show tensile lattice stress along the *c* axis, according to some reports.^22,23^ The asymmetry of the diffraction line indicates that the structural flaws at the substrate and A3ZO interface are the cause of the stress.

### 3.2. X-ray photoelectron spectroscopy (XPS) analysis

**Fig. 3:**
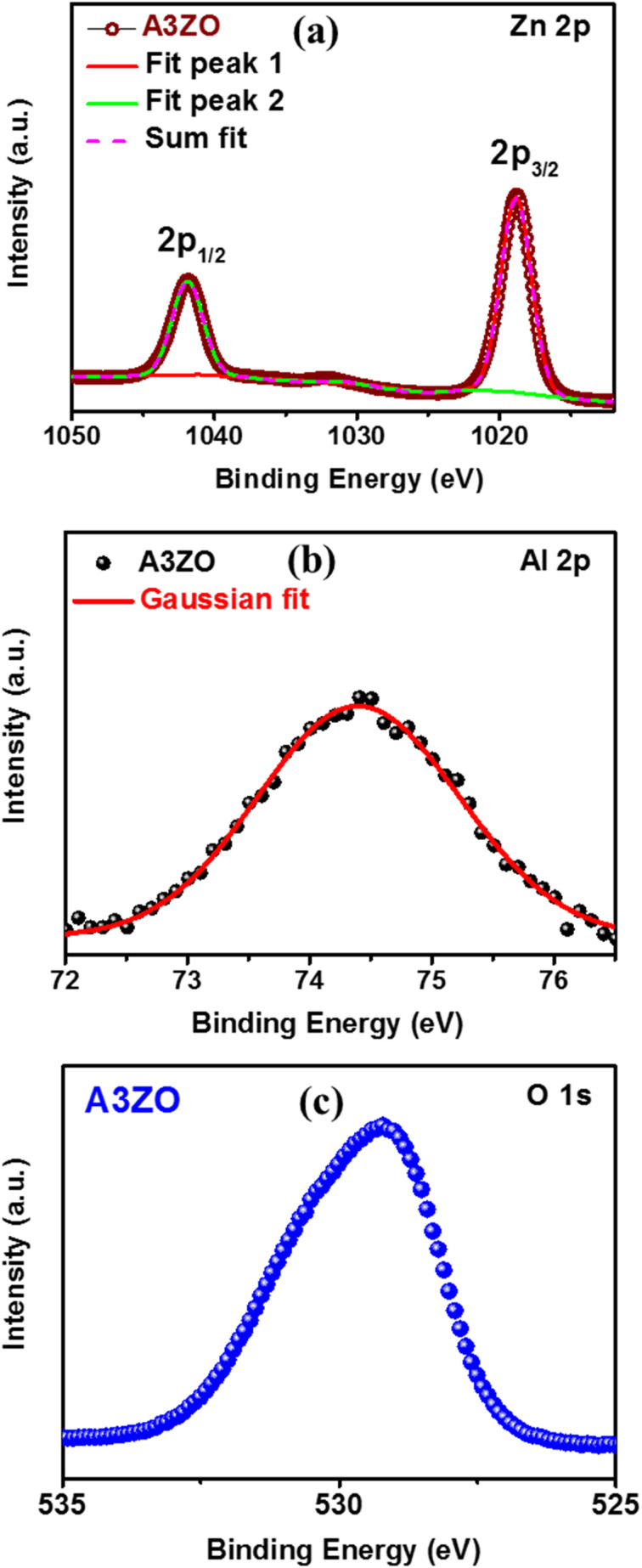
XPS spectra of (a) Zn 2p, (b) Al 2p, and (c) O 1s of the A3ZO NPs.

We conducted X-ray photoelectron spectroscopy (XPS) analysis to investigate the elemental chemical states and confirm the incorporation of aluminum (Al) during the sol–gel process. To mitigate specimen charging effects, binding energies obtained from the XPS analysis were referenced to the C 1s peak at 284.6 eV. In Fig. 3a, the core levels of Zn 2p_1/2_ and Zn 2p_3/2_ for the A3ZO nanoparticles were centered at 1041.8 eV and 1018.8 eV, respectively, exhibiting a spin–orbit splitting of 23 eV, consistent with other ZnO architectures.^12^Fig. 3b displays the Al 2p core level spectrum of the A3ZO nanoparticles, indicating a shift in the Al 2p peak with increasing Al concentration, reaching 74.3 eV. These observations provide compelling evidence for the successful integration of Al ions into the ZnO matrix. The ratio of Al 2p to Zn 2p areas was determined to be 0.035. The O 1s spectrum in Fig. 3c reveals a peak at 529.3 eV, attributed to oxidized metal ions on the nanostructure, such as O–Al and O–Zn, within the ZnO NPs. This suggests that Al doping significantly impacts the valence band state structure.

### 3.3. Morphological analysis

**Fig. 4:**
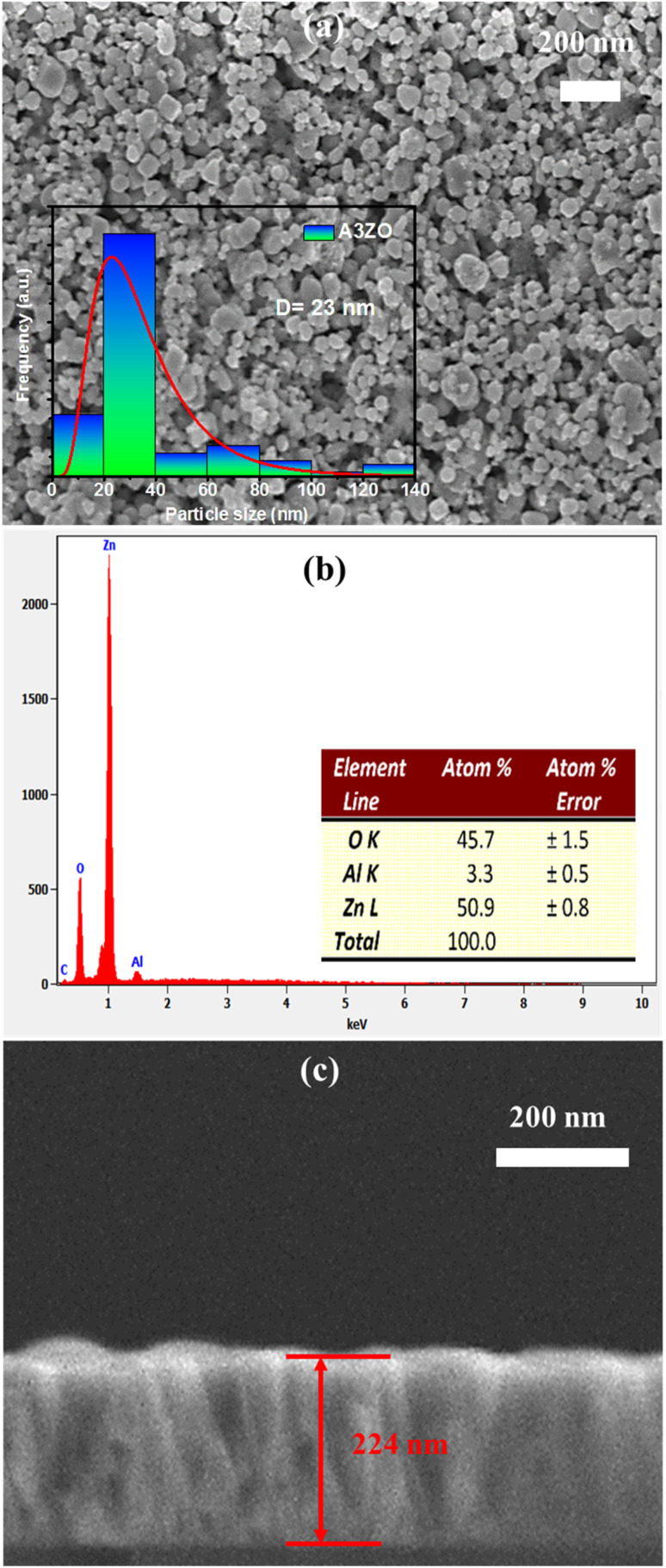
(a) SEM image of the prepared A3ZO nanoparticles with diagram of particles distributions (inset). (b) EDX spectrum of the prepared A3ZO NPs. (c) SEM image of the A3ZO/p-Si heterostructure.

We conducted scanning electron microscopy (SEM) analyses to examine the morphology of Al_3%_-doped ZnO (A3ZO) in both nanoparticle and thin film forms. In Fig. 4a, SEM images reveal spherical nanoparticles with an average size below 100 nm, confirming their nanoscale dimensions. Using ImageJ software, we estimated the grain size distribution, shown in the inset of Fig. 4a, which indicates a mean size of approximately 23 nm for A3ZO nanoparticles. Moreover, we studied the energy-dispersive X-ray spectroscopy (EDX) spectrum for the prepared A3ZO nanopowder, presented in Fig. 4b. The spectrum exhibits multiple peaks at varying intensities, corresponding to the elements Zn, O, and Al, with percentage values of 50.9%, 46.7%, and 3.3%, respectively. The presence of Al at approximately 3.3%, closely aligning with the intended 3% doping level, confirms the successful incorporation of the desired dopant. Additionally, the SEM image presented in Fig. 4c for the thin film indicates a thickness of 224 nm, as determined using ImageJ software.

**Fig. 5:**
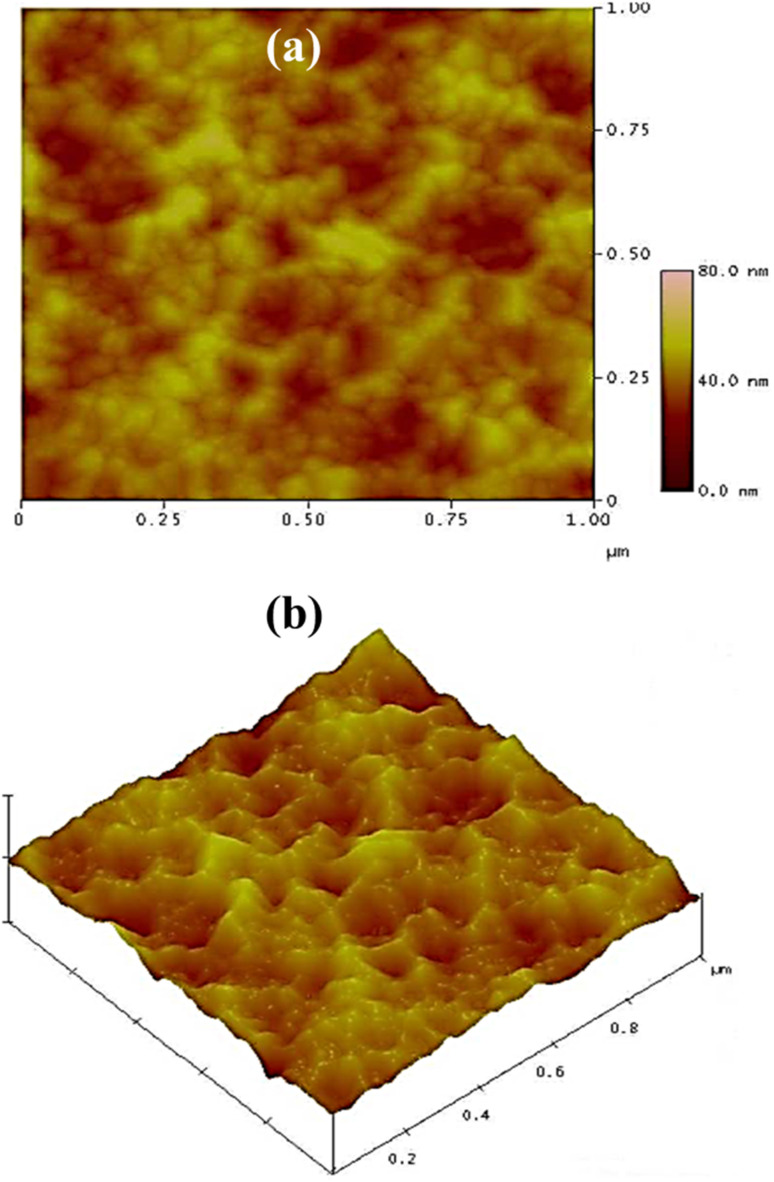
(a) The 2-D and (b) 3-D AFM of as-grown A3ZO thin film on glass substrate under the same growth conditions as the film on p-Si.

Fig. 5a and b show the 2-D and 3-D AFM of the A3ZO thin film as-grown on glass substrate under the same growth conditions as the film on p-Si, respectively. The surface film is composed of dense cone-like grains with evident outgrowths, resulting in an increased specific surface area and reduced surface reflection to external light.

### 3.4. Optical properties

**Fig. 6:**
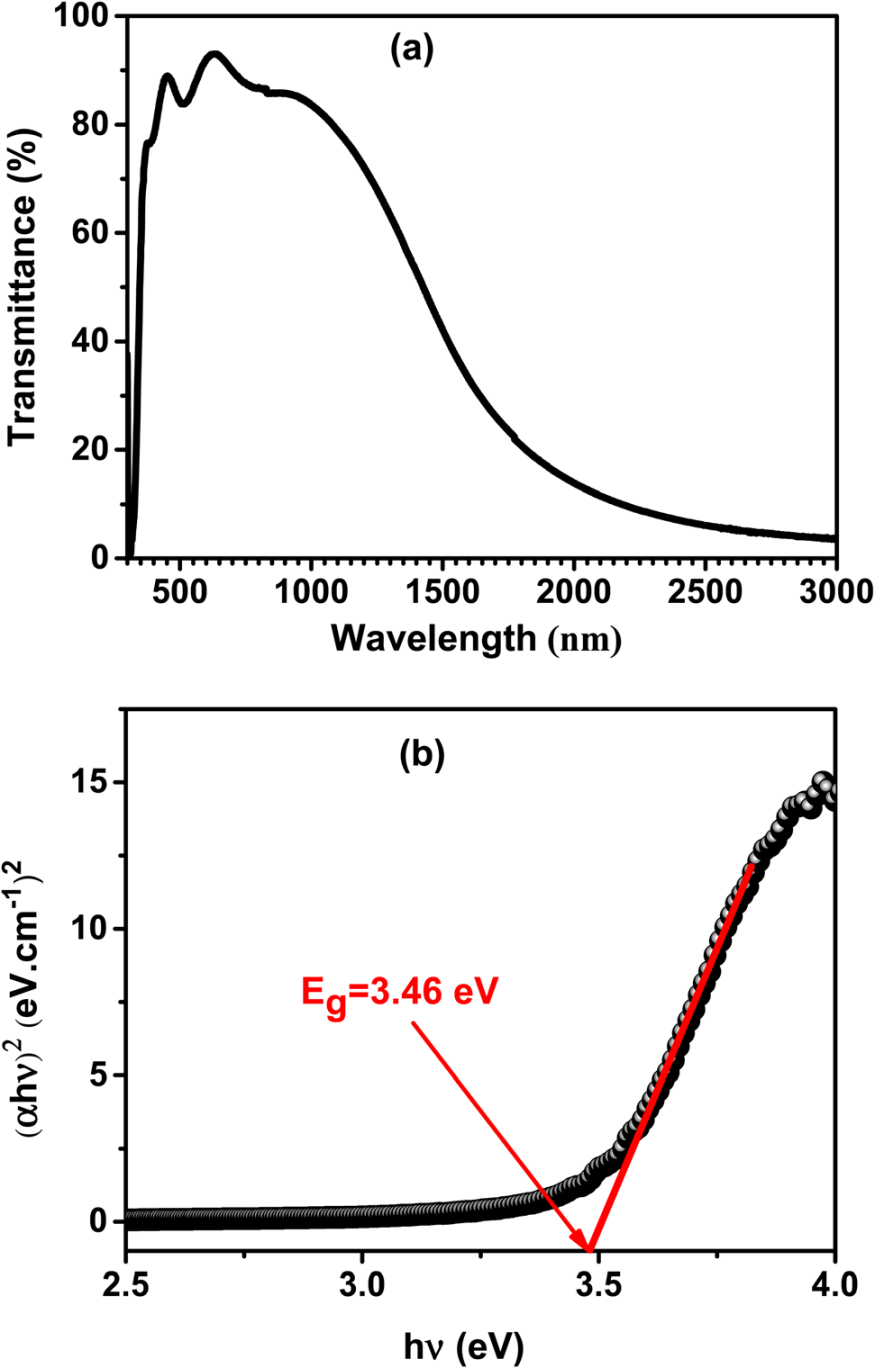
(a) Transmission spectrum of A3ZO film deposited on a glass substrate under the same growth conditions as the film on p-Si. (b) The plot of (*αhν*)^2^*versus hν*.

The optical properties of the deposited film were studied by the spectrum of transmission in the UV-visible-NIR region. The A3ZO film's transmission spectrum, deposited on a glass substrate under the identical fabrication conditions, is shown in Fig. 6a. The film has a high transmittance in the visible region, as seen in the figure. It may be inferred that when the junction is illuminated, most of the light can pass through the Al_3%_ doped ZnO film and reach the Si substrate which it can produce photo-generated carriers. (α*hν*)^2^ is displayed as a function of photon energy *hν*, where *h* is the Planck constant and *ν* is the photon frequency. This is depicted in the Fig. 6b. The absorption coefficient (*α*) is determined by using the formula *αd* = ln(1/*T*), where *d* and *T* are the film's thickness and transmittance, respectively. (α*hν*)^2^ is plotted as a function of photon energy *hν*, where *h* is the Planck constant and *ν* is the photon frequency. *α* is the absorption coefficient, calculated using *αd* = ln(*1*/*T*), *d* and *T* are the thickness and transmittance of the film, respectively.^24^ According to SEM image, the studied A3ZO thin film has a thickness of roughly 224 nm. Extrapolating the linear fit to the intersection of the *hν* axis will give the band gap (*E*_g_) value for the film. The *E*_g_ for the film is 3.46 eV, which is reasonably close to the theoretical value of zinc oxide (3.4 eV).^25^

### 3.5. Complex impedance analysis

**Fig. 7:**
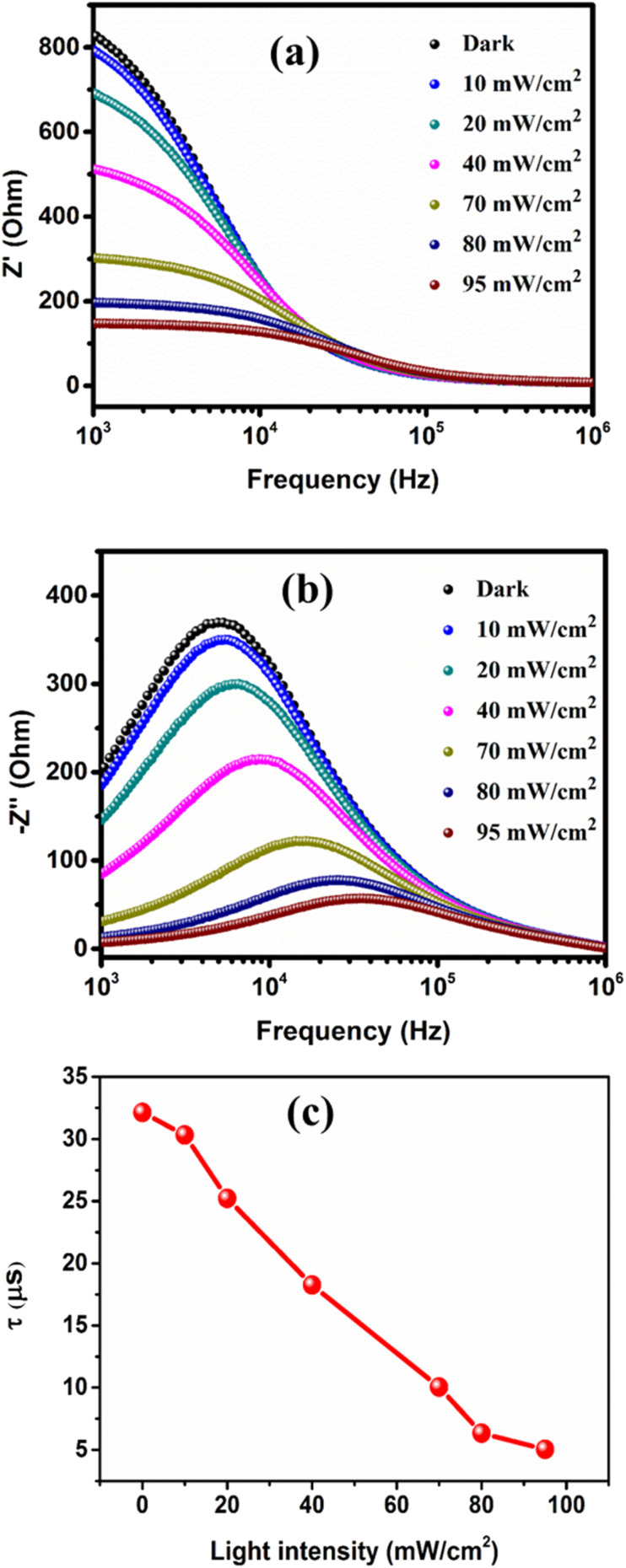
Frequency dependence of (a) the real part (*Z*′) and (b) the imaginary part (*Z*′′) of impedance at different light intensities. (c) Light intensity dependence of the relaxation time.

Fig. 7a and b illustrate the frequency dependence of the real part (*Z*′) and imaginary part (*Z*′′) of the complex impedance, respectively, for the studied heterojunction at various light intensities. Both *Z*′ and *Z*′′ of the impedance display a decreasing trend with an increase in light intensity. In Fig. 7a, high *Z*′ values form a plateau at low frequency, which can be attributed to total polarization resulting from space charge, dipoles, and electrons.^26,27^ Beyond this plateau region, the *Z*′ values decrease with an increase in frequency and eventually converge for all light intensities at higher frequency due to polarization weakening. In Fig. 7b, as frequency increases, *Z*′′ gradually increases and reaches a peak at a specific frequency (*f*_max_) and then declines with further increase in frequency. Moreover, with an increase in light intensity, the *Z*′′ peak reduces in magnitude and shifts towards a higher frequency range. This behavior of *Z*′′ suggests the existence of a dielectric relaxation process in the studied heterojunction, which arises from the separation of responses between capacitive and resistive ones.^28^ The significant broadening of the *Z*′′ peaks with increasing light intensity indicates the presence of an illumination-dependent electrical relaxation phenomenon in the studied heterojunction.^29^ Furthermore, both *Z*′ and *Z*′′ values converge at higher frequency, possibly indicating the accumulation of space charge in the device. The relaxation time (*τ*) of the heterojunction can be determined using the equation^30^

5 *τ* = 1/2π*f*_max_

Fig. 7c illustrates that the relaxation time decreases with an increase in light intensity. This suggests that the orientation of electrical charges in the interface is facilitated by illumination. A lower relaxation time (*τ*) under illumination indicates more efficient exciton dissociation at the heterojunction interface and a faster charge transport process, leading to an excellent responsiveness of the heterojunction.^31^Fig. 7b shows a slightly asymmetric curve, which may be due to the superposition of several peaks corresponding to the distributions of associated relaxation times. This implies that there are at least two electrical activity regions in the device, one of which can be represented by an RC parallel circuit.^32^

**Fig. 8:**
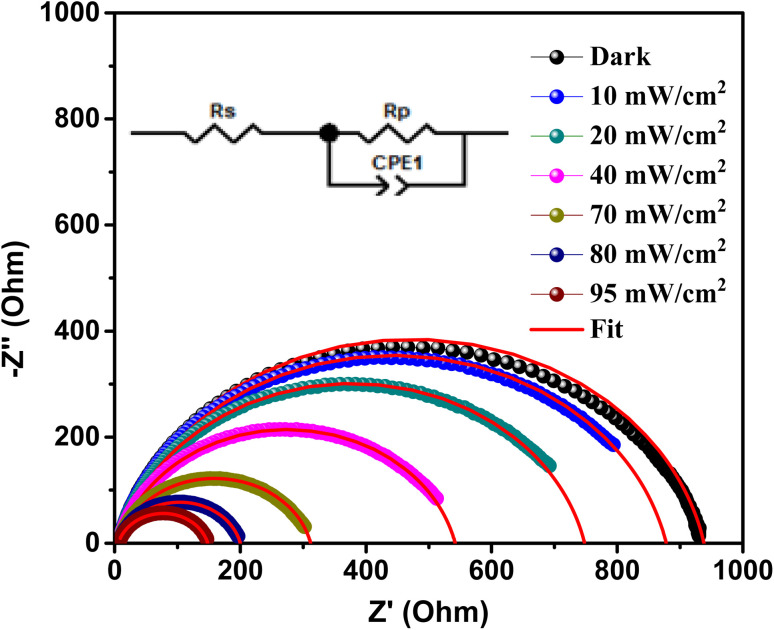
Nyquist impedance spectra of Au/A3ZO/p-Si/Al heterojunction and their corresponding fitting lines at different light intensities with the electrical equivalent circuit model (inset).

Based on the sample structure, it is important to note that there are two distinct activities occurring within the Au/Al doped ZnO/p-Si/Al heterojunction. In Fig. 8, we present the Nyquist impedance spectra of the A3ZO/p-Si heterojunction under varying light intensities. To elucidate and characterize these activities, we employed the “Zview” program to fit the experimental curves, represented by solid red lines, and the corresponding equivalent circuit is shown in the inset of Fig. 8.

The equivalent circuit model employed here serves as a powerful tool for understanding the underlying electrical behaviors of the heterojunction. Within this circuit, several key parameters are defined. *R*_p_ (parallel resistance) signifies the charge transfer resistance, which is a measure of the hindrance to charge transfer occurring at the interface between the A3ZO film and the Si substrate. CPE represents the constant phase element, which characterizes the capacity at this same interface, providing insights into the charge storage behavior.

Furthermore, within the circuit, *R*_s_ (series resistance) also plays a crucial role, representing the charge transfer resistance and capacity at the interface between the Si substrate and the bottom electrode. These parameters are vital in assessing the overall electrical performance of the heterojunction and can shed light on how the various components interact. The fitted values are presented in the Table 2.

**Table 2:** Fitting parameters from Nyquist diagram

Light intensity (mW cm^−2^)	Series resistance *R*_S_ (ohm)	Shunt resistance *R*_p_ (ohm)	*C*_PE_ (10^−9^ F)	*α*
Dark	8	930	120	0.88
10	8	870	125	0.87
20	8	740	130	0.87
40	7	535	135	0.86
70	7	305	157	0.86
80	7	193	159	0.86
95	6	142	170	0.85

It's noteworthy that as light intensity increases, we observe distinct effects on these circuit elements. The series and parallel resistances (*R*_s_ and *R*_p_) tend to decrease, indicating enhanced charge transfer efficiency at the interface between the A3ZO film and the Si substrate. This decrease in resistance is attributed to the photoexcitation of charge carriers, which facilitates their movement across the heterojunction.

Conversely, the capacitance represented by CPE increases with rising light intensities. This phenomenon can be attributed to the generation of additional charge carriers in response to increased illumination, leading to a higher capacity for charge storage at the A3ZO film-Si substrate interface. This change in capacitance is indicative of the enhanced charge accumulation and storage capabilities of the heterojunction under intensified illumination conditions.

### 3.6. Current–voltage characteristics

**Fig. 9:**
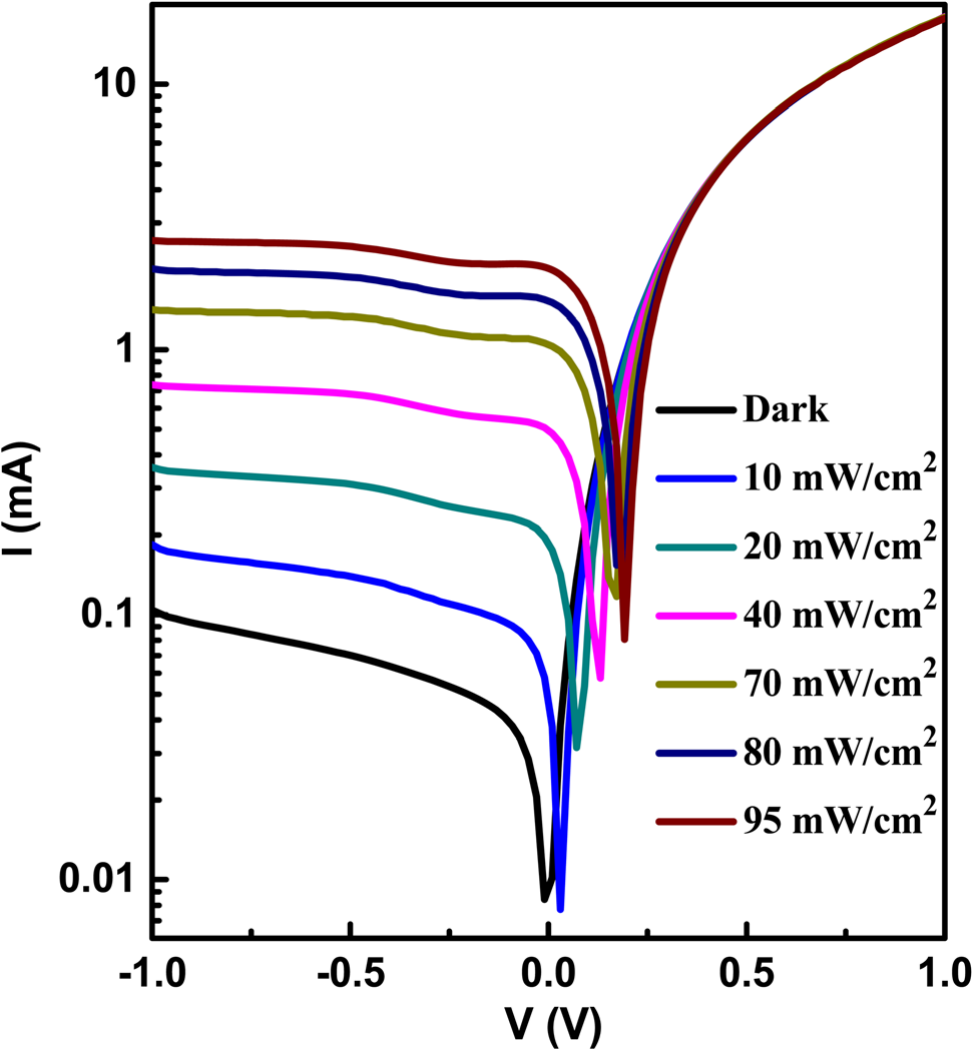
The semilogarithmic *I*–*V* curves of the prepared heterojunction at different light intensities.

Fig. 9 displays the semilogarithmic current–voltage characteristic of the Au/A3ZO/p-Si/Al structure at different light intensities. It is evident from Fig. 9 that the heterojunction exhibits clear diode behavior. Under dark conditions, the heterojunction has a large dark current at forward voltage and a very weak current at reverse voltage. At a bias voltage of +1 V, the forward dark current of the device was 17.98 mA, while the reverse leakage current was 0.10 mA at −1 V, resulting in a rectification ratio (*I*^+^/*I*^−^) of 179.8. This ratio suggests that a good p–n junction has been formed between the n-type A3ZO thin film and p-type Si substrate.^33^ The current increases with increasing light intensities at reverse voltage, while the increase is more pronounced. At reverse voltage, the current increases and eventually saturates after −1 V. The saturation voltage (−1 V) of the current remains essentially stable with an increase in light intensity. At a bias voltage of −1 V, the dark current (*I*_dark_) is 0.10 mA. Under a light intensity of 95 mW cm^−2^, the light current (*I*_light_) is 2.55 mA. Therefore, the photo current (*I*_photo_ = *I*_light_ − *I*_dark_) is 2.45 mA. The value of *I*_photo_ is 24.5 times that of *I*_dark_, indicating that the device exhibits good photosensing performance.

### 3.7. Mechanism research

**Fig. 10:**
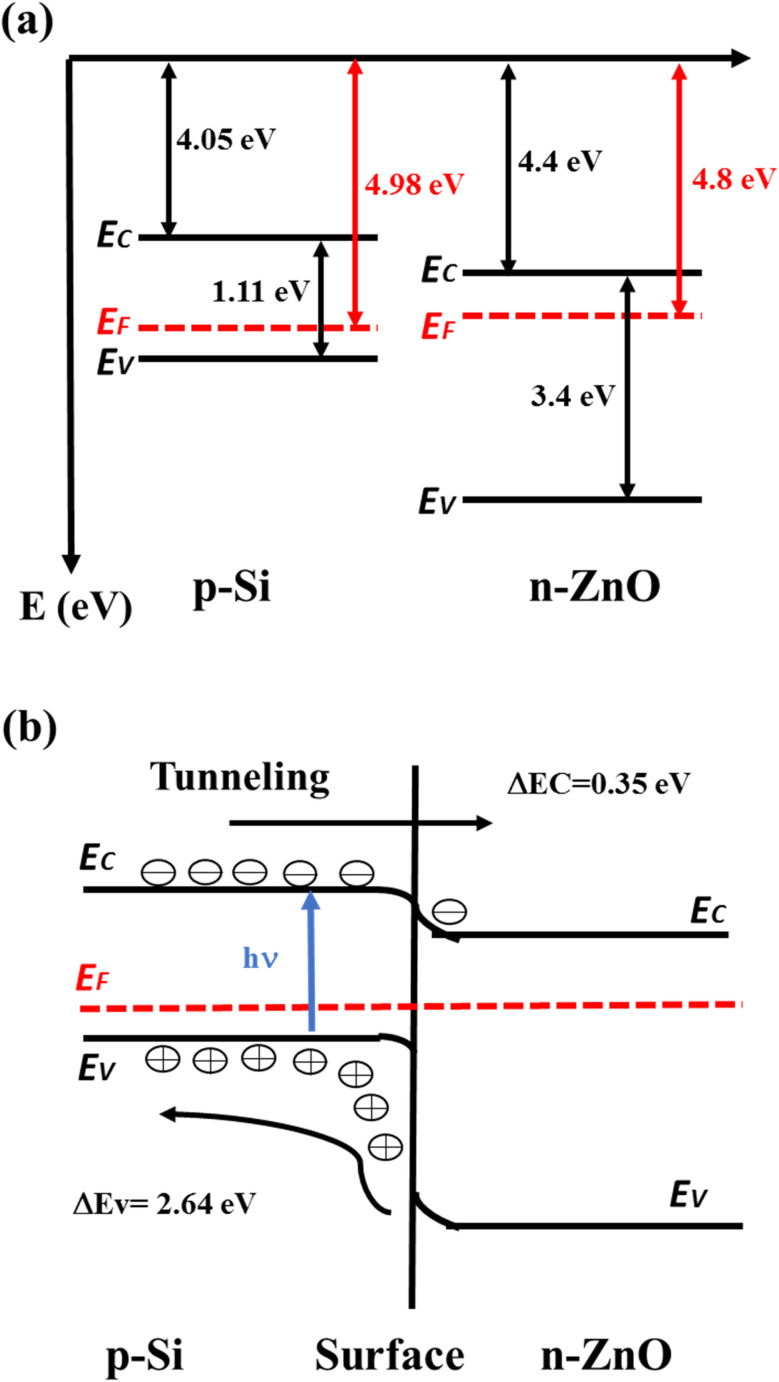
(a) The *E*_C_, *E*_g_, *E*_F_ and *E*_V_ values of p-Si and n-ZnO. (b) Energy band diagram of n-ZnO/p-Si heterojunction under illumination.

Conduction band edge (*E*_C_), Fermi Level (*E*_F_), and valence band edge (*E*_V_) of p-Si were calculated to be 4.05 eV, 4.98 eV, and 5.16 eV, respectively, based on the physics of semiconductors.^34^ The theoretically calculated values of *E*_C_, *E*_F_, and *E*_V_ for n-type ZnO provided by Nakamura *et al.*^35^ were 4.4 eV, 4.8 eV and 7.8 eV, respectively. From UV-vis spectrum analysis, the band gap of the A3ZO film was found to be 3.46 eV. Therefore, a schematic energy band diagram for the A3ZO film and p-Si was constructed, as shown in Fig. 10a. The Fermi level of the A3ZO film was found to be above the mid-gap, indicating good n-type semiconductor properties.^36^Fig. 10b shows the processes of photoexcitation and carrier transport in the heterojunction under illumination. The carriers in the n-i-p junction can pass the SiO_2_ layer by F–N tunneling. The photo-generated electrons in the n-i-p junction can tunnel through the SiO_2_ thin layer to the conductive band of n-A3ZO at the forward bias voltage. SiO_2_, as an insulating buffer, can decrease carrier recombination near the interface,^37^ which is beneficial to carrier transport. Under dark conditions, a forward bias voltage applied to the heterojunction (p-Si is the anode and n-A3ZO is the cathode) is less than the built-in potential. Most carriers cannot overcome the built-in potential, so there is no current in the circuit. When a forward bias voltage is greater than the built-in potential, conductive carriers and current continually increase with an increase in voltage. Under visible illumination, most visible incident light travels through the n-A3ZO thin film to the Si substrate. The photon energy absorbed by p-Si generates electron–hole (e–h) pairs. The photo-generated e–h pairs in the p-Si substrate are separated under the built-in electric field. The photo-generated electrons transfer from the valence band to the conduction band, while the photo-generated holes stay on the side of the p-Si substrate.^38^ The number of majority carriers before illumination is much higher than that of photo-generated e–h pairs; thus, the photo-generated e–h pairs have little effect on the majority carriers in the n-A3ZO film or Si substrate.^39^ Due to the F–N tunneling mechanism, the photo-generated electrons can tunnel through the SiO_2_ layer to the conduction band of the n-A3ZO in the 0.25–0.50 V range, which makes majority carriers of n-A3ZO increase and the Fermi level of n-A3ZO shifts upward. Therefore, the current increases slightly with an increase in the forward voltage. The photo-generated holes increase carrier concentration of p-Si, which lowers the Fermi energy level of p-Si. This effect makes the difference of Fermi levels between the n-A3ZO and p-Si decrease. When a reverse bias voltage is applied to the heterojunction (n-A3ZO is the anode and p-Si is the cathode), the minority carriers (hole carriers in n-A3ZO and electron carriers in p-Si) are conductive carriers, and the current is weaker.^40^ Under visible illumination, photo-generated electrons in the p-Si area participate in drift motion and spread to the p–n heterojunction at reverse voltage. The number of minority carriers increases significantly with an increase in light intensity. The reverse current increases under visible illumination, making the n-A3ZO thin film/p-Si demonstrate a good photosensing performance to visible light. Therefore, the current change of the heterojunction is larger at reverse.

## 4. Conclusion

In conclusion, this scientific paper has presented a comprehensive investigation on the synthesis, characterization, and electrical evaluation of the Au/Al-doped ZnO/p-Si/Al heterostructure under both dark and light conditions. The study successfully demonstrated the enhanced conductivity and optoelectronic performance of the fabricated heterostructure, highlighting its potential application in photovoltaic devices. The structural analysis confirmed the presence of a hexagonal wurtzite structure with (111) and (002) orientations, exhibiting a high degree of texture along the *c*-axis. Moreover, the current–voltage and impedance measurements provided valuable insights into the electrical behavior of the heterostructure. These findings contribute to the field by expanding the knowledge of advanced heterostructures for next-generation solar energy conversion. The research outcomes presented in this paper pave the way for further exploration and optimization of similar heterostructures, fostering the development of efficient and sustainable energy conversion technologies.

## Conflicts of interest

The authors affirm that they did not accept any money, grants, or other assistance for the creation of this manuscript.

## Supplementary Material
