# Correction: Light-enhanced electrical behavior of a Au/Al-doped ZnO/p-Si/Al heterostructure: insights from impedance and current–voltage analysis

**DOI:** 10.1039/d3ra90100a

**Published:** 2023-10-26

**Authors:** Majdi Benamara, Kais Iben Nassar, Sonia Soltani, Afef Kallekh, Ramzi Dhahri, Hassen Dahman, Lassaad El Mir

**Affiliations:** a Laboratory of Physics of Materials and Nanomaterials Applied to the Environment, Faculty of Sciences of Gabes, University of Gabes Erriadh 6079 Gabes Tunisia majdibenamara1@gmail.com; b Department of Physics, I3N-Aveiro, University of Aveiro 3810-193 Aveiro Portugal; c Department of Physics, College of Science and Arts, Qassim University Dariyah 58251 Saudi Arabia; d Department of Mathematics, College of Science and Art Muhyl Assir, King Khalid University Abha 61413 Saudi Arabia; e Department of Physics, Faculty of Sciences and Arts, Najran University P.O. Box 1988 Najran 11001 Saudi Arabia

## Abstract

Correction for ‘Light-enhanced electrical behavior of a Au/Al-doped ZnO/p-Si/Al heterostructure: insights from impedance and current–voltage analysis’ by Majdi Benamara *et al.*, *RSC Adv.*, 2023, **13**, 28632–28641, https://doi.org/10.1039/D3RA06340B.

The authors would like to clarify the affiliation list of the published article to correctly show where the work was conducted. In the original article, the affiliation at the time of publication of one author (Ramzi Dhahri) was listed as Laboratory of Physics of Materials and Nanomaterials Applied to the Environment, Faculty of Sciences of Gabes, University of Gabes, Erriadh, 6079, Gabes, Tunisia. However, this author also had a second affiliation which was regretfully omitted: Department of Physics, Faculty of Sciences and Arts, Najran University, P.O. Box 1988, Najran, 11001, Saudi Arabia. The corrected list of affiliations at the time of publication of the original article is presented here.

The Royal Society of Chemistry apologises for these errors and any consequent inconvenience to authors and readers.

## Supplementary Material

